# Crossing the membrane—What does it take to flip a phospholipid? Structural and biochemical advances on P4-ATPase flippases

**DOI:** 10.1016/j.jbc.2024.107738

**Published:** 2024-09-02

**Authors:** Kadambari Vijay Sai, Jyh-Yeuan (Eric) Lee

**Affiliations:** Department of Biochemistry, Microbiology and Immunology, Faculty of Medicine, University of Ottawa, Ottawa, Ontario, Canada

**Keywords:** lipid transport, P4-ATPase, membrane protein, membrane structure, phospholipid, sphingolipid, phospholipid vesicle, cryo-electron microscopy, X-ray crystallography

## Abstract

Membrane asymmetry is critical for maintenance of several different processes such as cell signaling, apoptosis, and vesicular transport in various eukaryotic systems. Flippases of the P4-ATPase family are associated with flipping phospholipids from the luminal or exoplasmic leaflet to the cytosolic leaflet. P4-ATPases belong to the P-type ATPase family, which are activated by phosphorylation and couple ATPase activity to substrate translocation. These proteins possess a transmembrane domain responsible for substrate transport, while the cytosolic machinery performs the necessary ATP hydrolysis for this process. Several high-resolution structures of human or yeast P4-ATPases have recently been resolved, but a comprehensive overview of the changes for reaction cycle in different members was crucial for future research. In this review, we have compiled available data reflecting the reaction cycle-associated changes in conformation of P4-ATPases. Together, this will provide an improved understanding of the similarities and differences between these members, which will drive further structural, functional, and computational studies to understand the mechanisms of these flippases.

The transbilayer asymmetry of phospholipids is critical for several different processes such as cell signaling, initiation of apoptosis, vesicle formation and transport, regulation of membrane characteristics, blood coagulation, and host–virus interactions in several eukaryotic systems (1). In eukaryotes, this is primarily maintained by three different classes of membrane proteins: flippases, floppases, and scramblases. Flippases, from the P4-ATPase family, flip phospholipids from the exoplasmic leaflet to the cytosolic leaflet (2), while floppases from the ATP-binding cassette superfamily transport them from the cytosolic leaflet to the exoplasmic leaflet in ATP-dependent processes. However, scramblases, like Xrk8, nondiscriminately transfer phospholipids in both leaflets’ directions independent of ATP (3). P4-ATPases belong to the larger P-type ATPase family, which are activated by phosphorylation at a conserved aspartate residue (in a DKTGT motif) and couple ATPase activity to substrate transport. The P1-, P2-, and P3-type ATPases, present in prokaryotes and eukaryotes, transport cations and other small, charged substrates (4). However, P4- and P5-ATPases are unique to eukaryotes. The substrates of P5-ATPases are currently known to transport polyamines, peptides, and polypeptides, but their substrate specificity is still being uncovered (5). P4-ATPases are distinct in this group due to their ability to transport phospholipids such as phosphatidylcholine (PC), phosphatidylserine (PS), phosphatidylethanolamine, and sphingolipids, such as glucosylceramide (GlcCer) and sphingomyelin (6).

P4-ATPases generally possess the same overall architecture as the P-type ATPases (7) but are predominantly heterodimeric proteins, with a catalytic α-subunit and an accessory β-subunit (Fig. 1). The former comprises of a transmembrane (TM) domain with 10 helices, and the cytosolic nucleotide-binding domain (N-domain), phosphorylation domain (P-domain), and actuator domain (A-domain) (8). Further, the terminal regions of these proteins may also function as a regulatory domain (R-domain) (9). The β-subunit, on the other hand, is comprised of a TM domain with two helices and an exoplasmic domain (10).

**Figure 1:**
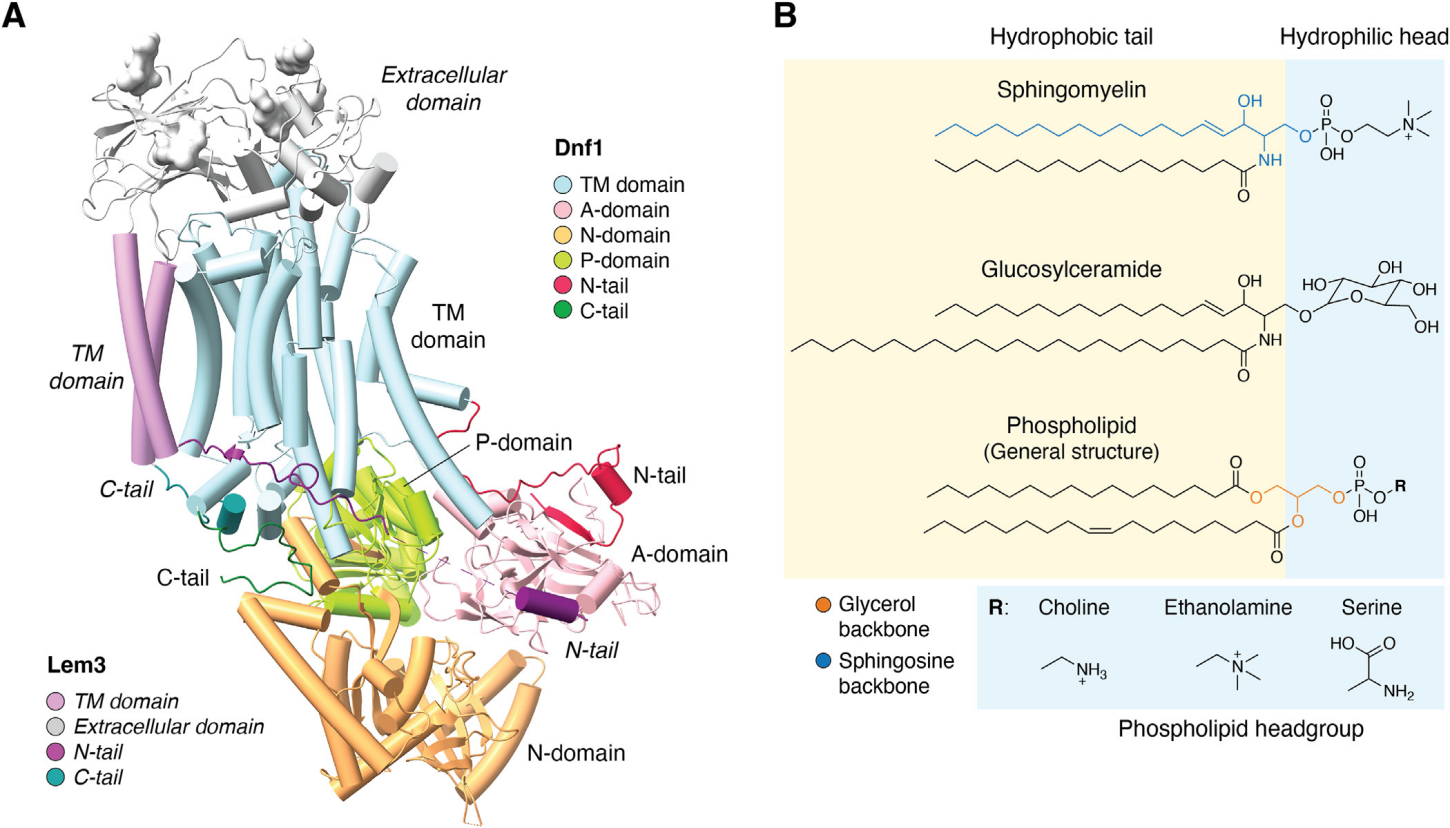
**Structure and substrates of P4-ATPases.***A*, general structure of a P4-ATPase. A, The α-subunit (here, Dnf1, PDB 7KYC) is composed of three cytosolic domains—the nucleotide-binding (N) domain (*light orange*), the phosphorylation (P) domain (*light green*), and the actuator (A) domain (*light pink*)—and a transmembrane (TM) domain (*light blue*). Similarly, the β-subunit (Lem3) also consists of a TM domain (*light purple*) and an exoplasmic domain (*gray*) with glycosylation (visible as *blobs*). Notably, the N- (Dnf1, *crimson*; Lem3, *magenta*) and C-tails (Dnf1, *dark green*; Lem3, *teal*) of both subunits are cytosolic. The cytosolic domains are mainly associated with the reaction cycle, while the TM domain performs the substrate transport function. The β-subunit is mainly associated with stabilization of the α-subunit. (This color scheme will be maintained). *B*, known substrates of P4-ATPases. Most phospholipids are amphipathic due to the presence of both hydrophobic (*yellow*) and hydrophilic (*blue*) regions. They possess a glycerol backbone (*solid line*), one or two hydrophobic fatty acid tails, and a hydrophilic phosphate headgroup, with variations in the alkyl chain (R group, *dashed line*). Sphingolipids, however, possess a sphingosine backbone (*dotted line*), with a hydrophobic fatty acid. Most characterized P4-ATPases are known to transport phospholipids such as phosphatidylcholine, phosphatidylserine, phosphatidylethanolamine, or sphingolipids such as sphingomyelin or glucosylceramide, in addition to other members of these families. Substrate specificities of each flippase can be found in Table 1.

P4-ATPases are divided into three subclasses, the P4A, B, and C. P4A and C are heterodimeric, while P4B are monomeric, lacking a β-subunit. P4C-ATPases are an intermediary group but appear to be evolutionarily closer to the P4A-ATPases (11). The most widely studied systems of P4-ATPases are the budding yeast, *Saccharomyces cerevisiae, Homo sapiens,* and *Arabidopsis thaliana*. *S.cerevisiae* has five members (Dnf1-3, Drs2, and Neo1), *H.sapiens* have 14 (ATP8A1, ATP8A2, ATP8B1, ATP8B2, ATP8B3, ATP8B4, ATP9A, ATP9B, ATP10A, ATP10B, ATP10D, ATP11A, ATP11B, and ATP11C), and *A.thaliana* has 12 (ALA1-12). While most of these belong to the P4A group, ScNeo1, HsATP9A, and HsATP9B belong to the P4B group, while AtALA2 belongs to the P4C group. Notably, the P4B group is absent in plants, while the P4C group is found almost exclusively in plants (with a few members in fungi), suggesting a possibility of these subclasses performing similar physiological roles in their respective systems (11).

As of now, the structures of some human and yeast P4-ATPases have been resolved, while several others have been examined using biochemical and biophysical approaches (8, 12, 13, 14, 15, 16, 17, 18, 19, 20, 21, 22, 23). We are hereby highlighting the current understanding of P4-ATPases from the structural perspective. [Other reviews have also attempted to compile the available cellular, biochemical, and structural information on P4-ATPases (6, 24, 25, 26) and P-type ATPases (27, 28)]

## Regulation of P4-ATPase activity and localization

The cytosolic terminal regions of P4-ATPases exhibit low sequence conservation. They have been implicated in two main functions—regulation of activity and trafficking/localization.

### Trafficking/localization

The roles of the terminal regions have been extensively described previously (9). The terminal regions of some P4B-ATPases and the human ATP10 and ATP11 proteins hold trafficking signals (29). ATP8A1, ATP10B, and ATP11C also contain motifs reminiscent of a dileucine motif associated with sorting in other TM proteins (30). In all these cases, the two leucines were found to be critical for their localization, but the significance of other residues varied (29, 31, 32). The LLxY motif in the C-tail of ATP11C is required to interact with the actin-binding protein ezrin, an essential molecule to stabilize the polarized plasma membrane localization in the motile breast cancer cell line, MDA-MB (33, 34).

### Regulation of P4-ATPases by self and external regulatory mechanisms

**Figure 2:**
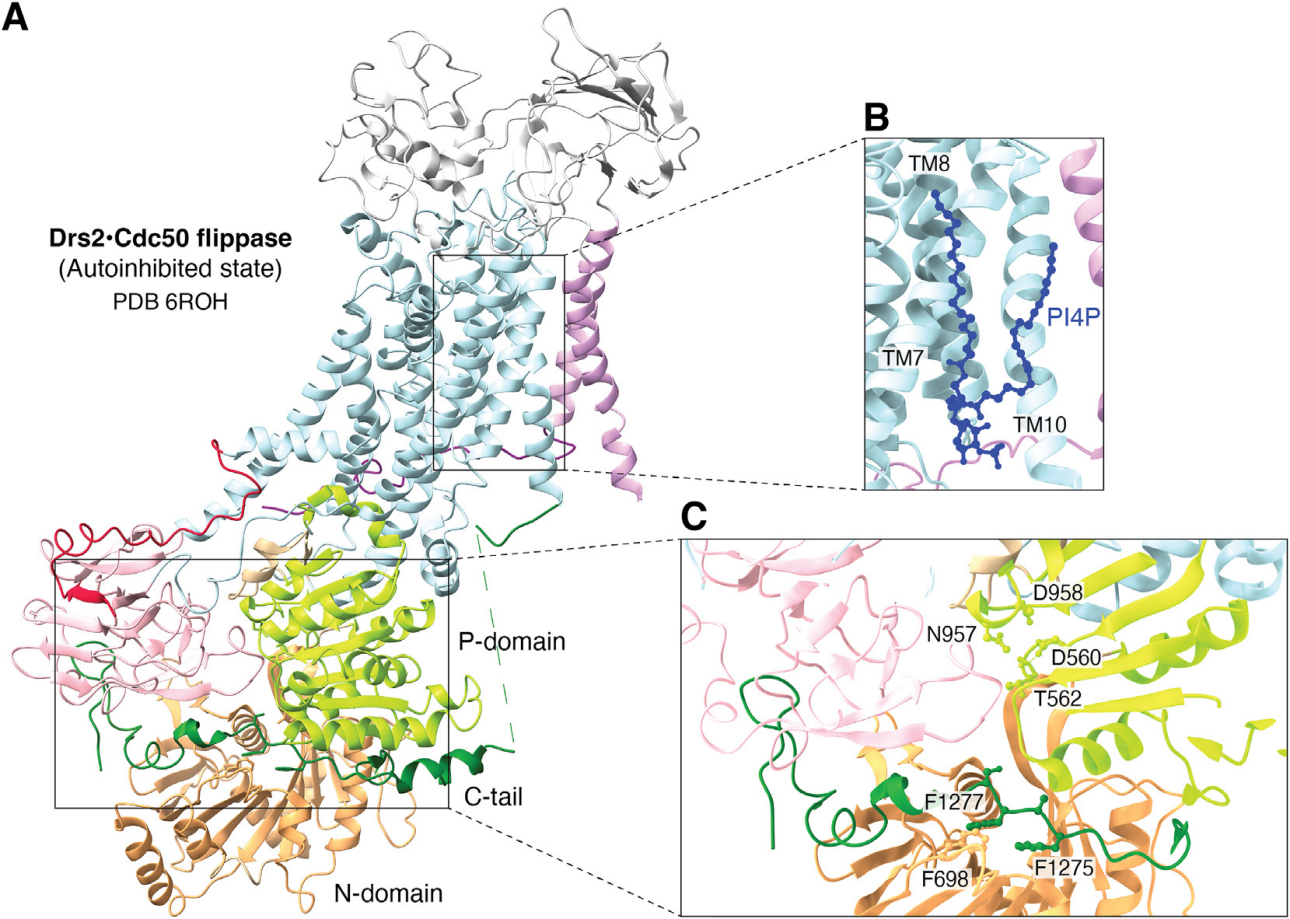
**General structure of an autoinhibited flippase Drs2-Cdc50.***A–C*, the C-tail (*green*) of Drs2 (PDB: 6ROH) is the primary determinant of autoinhibition of the flippase. It is this effect through its interactions with the cytosolic regions of the protein, through F1275 and F1277 of the GFAFS motif. This region of the tail is found at the interface of the A, N, and P domains. This prevents ATP from bridging the N- and P-domains at the interface shown in (*C*). Notably, PI4P binding as shown in (*B*) (PDB: 6PSX) between TM7, 8, and 10, along with C-terminal truncation is found to relieve autoinhibition *in vitro*. The exact mechanism of autoinhibition release *in vivo* is yet to be determined. In addition to the C-tail, the N-tail (*crimson*) also exerts a supporting role in autoinhibition by interacting with the A- (*light pink*) and P-domains (*light green*). PI4P, phosphatidylinositol-4-phosphate; TM, transmembrane.

The autoinhibitory domain, also known as the regulatory or R-domain, upon removal generally leads to activation of the flippase activity of the protein. The terminal regions interact with the cytosolic regions to autoinhibit the protein (Fig. 2*A*) (16, 35, 36). In ATP8B1 and Drs2, the terminal regions cooperate at the interface of the cytosolic domains to hinder ATP binding and phosphorylation through a C-terminal G(F/Y)AFS motif (15, 20, 21, 35) (Fig. 2*C*), while the C-tail of Dnf1 interacts with the cytosolic regions to regulate reaction cycle progression (12). The C terminus of ATP8A1 has a stabilizing effect (8), while that of ATP8A2 has a complex regulatory mechanism with regions responsible for autoinhibition and autoinhibition relief (37). C-terminal phosphorylation has also been identified as regulatory in some members (12, 20, 37, 38).

Although the C terminus is the primary regulatory region in the ATP8 proteins, it is short and disordered in those where the N-tail forms the R-domain. Meanwhile, the N-terminal regions of ATP11C contain three caspase recognition sites, cleavage of which affects N-domain folding, leading to PS exposure and ultimately, apoptosis (22).

Phosphoinositides, especially phosphatidylinositol-4-phosphate (PI4P), alleviate the autoinhibition of Drs2 through their interaction with the C-terminal domain of the protein (39). An Arf-like protein, Arl1, interacts with the N-terminal regions of Drs2 and the guanine nucleotide exchange factor Gea2p to form a complex, which is essential for proper functioning of Drs2 to maintain lipid asymmetry (39, 40). Arl1 is a GTPase which is involved in regulation of membrane traffic, while Gea2p is a GEF, typically involved in exchange of GDP for GTP. Together, this ternary complex is involved in vesicle budding (41). PI4P binding to a cavity between TM7, 8, and 10 allosterically relieves autoinhibition by removing the C-tail from the interface of the cytosolic domains (Fig. 2*B*). This is potentially followed by C-tail–Gea2p interaction (15, 16), since the Gea2p binding site on the C-tail is normally inserted within the cytoplasmic domains but is freed upon activation by PI4P. However, the exact mechanism of the expulsion and prevention of automatic re-autoinhibition by the C-tail is yet to be ascertained (16). Of note, another C-terminal basic patch has also been identified for PI4P binding (39), but it has yet to be structurally captured.

For ATP8B1, C- and/or N-terminal truncation is not sufficient for activation for PC flipping while it is sufficient to enhance the PS-stimulated ATPase activity (20, 21). Two independent groups identified different activators of this enzyme for lipid flippase activity. Phosphoinositides, especially PI(3,4,5)P_3_, increased PC-dependent activity, while cholate, especially tauroconjugated bile acids increased PS-dependent activity (20, 21). The PI(3,4,5)P3 binding site was narrowed down to either the TM domain or N terminus (20) and was recently observed in a cavity between TM7, 8, and 10 (like PI4P in Drs2) (42), while bile acid-binding site was found to be an extension of the P-domain, termed the P-loop (21). Since PI(3,4,5)P_3_ is expressed at very low levels in mammalian cells (43), it hints at tight control *in vivo*, potentially due to the natural abundance of PC in the exoplasmic leaflet. While the effects of PI(3,4,5)P_3_ with different substrates have been demonstrated (42), any potential interactions with bile acids and their impact on substrate transport is yet to be assessed. Notably, PI(3,4,5)P3 was found to be bound stably in the same cavity, albeit with partially flexibility, throughout the reaction cycle of ATP8B1 (42). The influence of the membrane composition on P4-ATPase structure and function was also observed when Dnf1 reconstituted in a nanodisc, a membrane mimetic system (13) as discussed in Section 6.

## Coupling of cytosolic machinery to reaction cycle

P4 and P5-ATPases couple substrate transport to the dephosphorylation phase of the cycle, while the P1-3 ATPases couple substrate transport to both the phosphorylation and dephosphorylation phases (17, 44). Additionally, the latter generally undergo huge rearrangements of their TM domains over the course of the cycle, but the resolved P4-ATPase structures have exhibited virtually superimposable TM domains throughout the process, with most of the reaction cycle-associated changes seen in the cytosolic domains (8). Previously, the rigidity of the TM domain was attributed to the β-subunit (8), but recent cryo-EM structures of Neo1 revealed that the uniformity of the TM domain was preserved even in the absence of a β-subunit (18).

**Figure 3:**
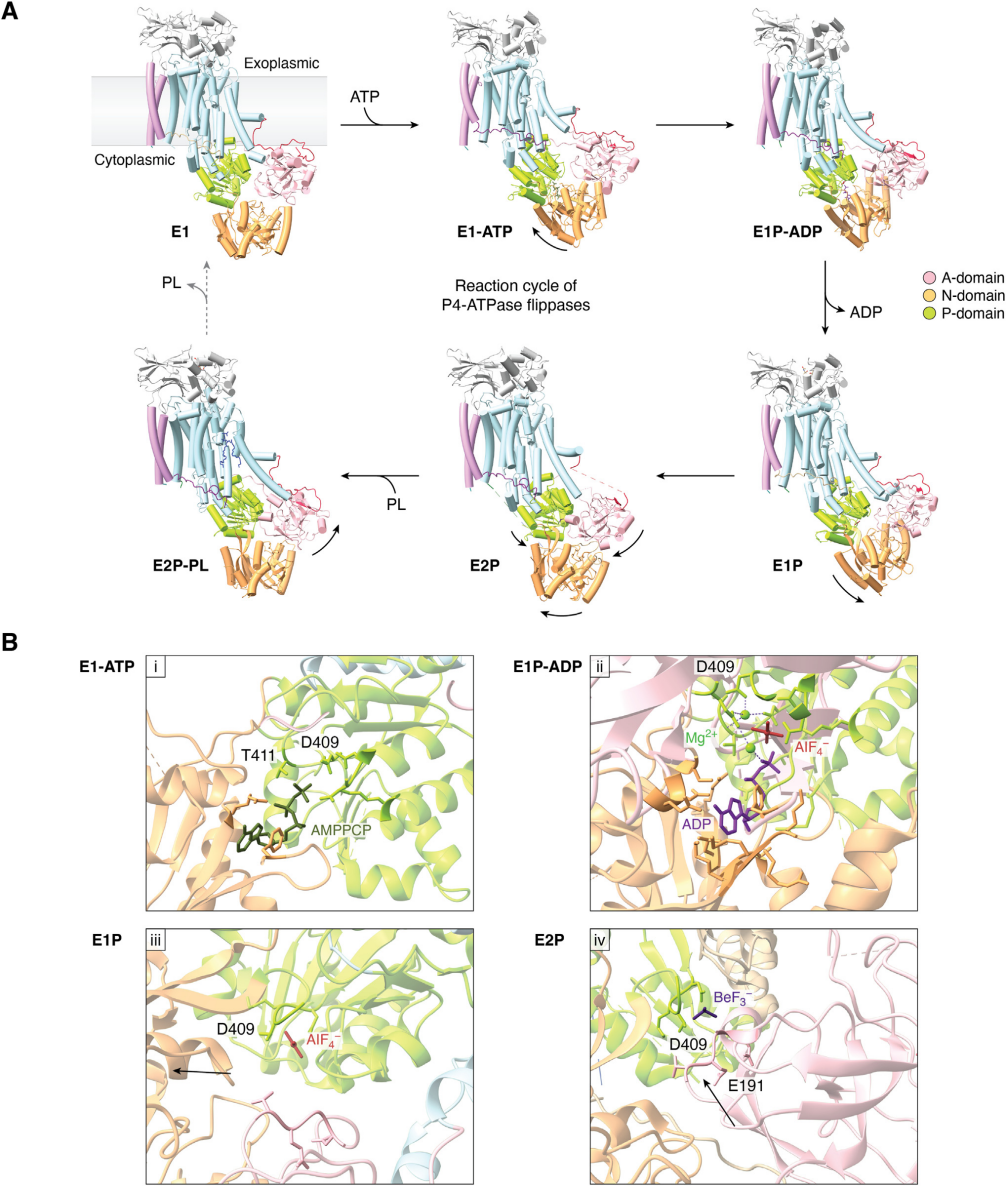
**Catalysis-driven structural changes of P4-ATPases.***A*, reaction cycle of P4-ATPases. The reaction cycle comprises two energy states—an inward open E1 state and an outward open E2 state—with several intermediate states of substrate binding and occlusion. The enzyme is released from its apo state (PDB: 6K7H) by the binding of ATP to the N-domain (*light orange*), and ATP bridges the N and P domains (*light green*) to form the E1-ATP state (PDB: 6K7J). Subsequently, the P-domain is phosphorylated at the conserved DKTG motif, forming the E1P-ADP state (PDB: 6K7K). Following ADP release, the transient E1P phosphoenzyme (PDB: 6K7N)) is created. In order to guard against spontaneous dephosphorylation by water, the A-domain (*light pink*), particularly the DGET motif, moves toward the P-domain, opening the exoplasmic gate through the linked TM1 and 2, thus creating the outward open E2P phosphoenzyme state (PDB: 6K7L). This allows substrate phospholipid (*dark blue*) binding on the exoplasmic side creating the E2Pi-PL state (PDB: 6K7M). As the phospholipid headgroup moves through the TM domains, it gets buried or occluded within the membrane due to accompanying closure of the TM 1 to 2 helices and A-domain movement in this stage. This subsequently opens the cytoplasmic gate, preparing the enzyme for substrate release into the cytoplasmic side. The *gray dotted arrow* for this transition indicates that the intermediates involved in substrate release have yet to be captured structurally. *B*, molecular interactions in the cytosolic regions associated with reaction cycle progression, as captured using ATP and phosphate analogs. (i) ATP binding at the interface of N- and P-domains, near the D409 and T411 of the DKTG motif of the P-domain is mimicked by the nonhydrolyzable ATP analog, adenosine-5'-[(β,γ)-methyleno]triphosphate (AMPPCP, *olive green*). The phosphate analog, AlF_4_, along with ADP, creates the E1P-ADP state, where the DKTG motif is phosphorylated, coordinated by Mg^2+^(*green sphere*), but ADP is yet to be released (ii), while on its own, it creates the E1P-state where the enzyme is phosphorylated, but the A-domain is still separated from the P-domain (iii). Finally, the phosphate analog, BeF_3_, brings the A-domain closer to the P-domain and bridges them through the DKTG–DGET interaction which is characteristic of the E2P state (iv). Residue numbers are based on ATP8A1. TM, transmembrane.

P4-ATPases, like other P-type ATPases, follow the Post-Albers reaction cycle (45, 46), involving a phosphoenzyme with two states, E1 and E2, depending on which face of the membrane they are accessible to (45, 46, 47). This involves several intermediate stages based on nucleotide and substrate binding (Fig. 3*A*).

### Apo-E1 state

This state (Fig. 3*A*), representing the native state of the enzyme, free from ligands, has been resolved for Dnf1/2, Drs2, and ATP8A1. The enzyme in the apo-state exhibits the characteristic stable TM domain and P-domain, but with an extremely flexible N- and A-domain (8). In autoinhibited members, this state is maintained by the association of the C-terminal G(F/Y)AFS motif with the cytosolic N-, P-, and A-domains (16).

### E1-ATP state

The enzyme is released from the inactive apo-state by binding ATP (Fig. 3*A*). This state is captured using the nonhydrolyzable form of ATP, AMPPCP. The ATP molecule is initially bound by a conserved phenylalanine of the N-domain through the adenine ring and then bridges N and P-domains, stabilizing them (Fig. 3*B* (i)). Here, the phosphate group of ATP is bound by the Asp and Thr of the conserved DKTGT motif, a conserved Asn and Asp of the P-domain and coordinated by an Mg^2+^ ion (8).

The strength of the interaction between the N-domain and A-domain varies between P4-ATPases in this state, but the A-domain flexibility is usually a common feature (17). However, Neo1 possesses a stable A-domain in the E1-ATP state. Although, A-domain stabilization usually involves the A–P domain interaction through the conserved DGET and DKTGT motifs, respectively, here it interacts with a helix-turn-helix motif of the N-domain, which could be responsible for the observed stability (18).

In Drs2, the PI4P binding induced autoinhibition relief (15) allows the A- and P-domains to move, resulting in exoplasmic gate closure due to movement of the connected TM1–2/TM3–6 and a state primed for ATP binding (16).

### E1P-ADP state

Subsequently, the molecule shifts to the almost identical E1P-ADP (Fig. 3*A*) state by phosphorylation of the invariant aspartate (mimicked by AlF_4_^−^) of the DKTGT motif and release of the terminal phosphate (Fig. 3*B* (ii)). The released phosphate is coordinated by an Mg^2+^ ion and the surrounding polar residues (16). TM2, which is connected to the A-domain is positioned between TM4 and TM6 at this stage, sealing the exoplasmic gate of the substrate translocation pathway (19). During phosphorylation, A-domain is flexible and moves outward, away from the N- and P-domains (8). The A-domain is positioned differently in P4-ATPases compared to the calcium transporter sarcoendoplasmic reticulum calcium ATPase, a change suggested to be due to a P4-specific helix-turn-helix motif in the N-domain. This favors the small dephosphorylation-associated movement, a characteristic feature of P4-ATPases (19).

### E1P state

Following the release of ADP, the N-domain becomes flexible again, which in turn causes the P-domain to orient itself toward the membrane, in preparation for the E2P state. The formation of the E1P state (Fig. 3*A*) from the E1-ADP state has been suggested requiring the largest energy change due to the hydrolysis of ATP (48). Meanwhile, the A-domain is positioned similar to the E1-ADP state (19). This state finds the phosphate analog exposed to bulk water and thus must be covered quickly to prevent dephosphorylation, hence making it a transient state (19) (Fig. 3*B* (iii)). The phosphorylation of P-domain orients it toward the TM domain but does not affect the connected TM4 due to a P4-specific insert in the TM4–P domain linker. The absence of this insert in other P-type ATPases such as the P2-ATPases allows coupling of TM4-dependent ion transport to the phosphorylation part of the cycle (17).

### E2P state

The E2P state (Fig. 3*A*) is formed by the movement of the dephosphorylation loop, particularly the DGET motif, of the A-domain, to cover the phosphorylated aspartate of the DKTGT motif, thus preventing spontaneous dephosphorylation. In this state, the A-domain, through DG of the DGET motif, is tightly bridged with the P-domain and thus is stable. Notably, the aspartate is unique to P4-ATPases and is speculated to be involved in controlling dephosphorylation through extensive stabilization by surrounding residues (11, 17). Since the N–P-domain bridging ADP has diffused away, the N-domain is separated from the P-domain. The phosphate analog BeF_3_^−^ is bound to the DKTGT motif in coordination with the Mg^2+^ ion, thus resembling the aspartylphosphate of the E2P state (8) (Fig. 3*B* (iv)). The TM domain remains unchanged until this stage. The movement of the A-domain, along with the N-terminal of the Cdc50 and C-terminal end of the TM4, stabilizes the TM4-facing P-domain (17). ATP9, ATP10A, and ATP11 proteins can generally leave the endoplasmic reticulum (ER) at the E2P stage, with the heterodimers associating with the β-subunit at this stage for stabilization (49).

The E1 and E2 states appear similar for P4-ATPases, whereas cation transporting P-type ATPases have notable changes in the A-domain. The A-domain of P4-ATPases traces a shorter path, pivoting about the dephosphorylation loop during the E1P-to-E2P transition. This limited movement has been attributed to the presence of conserved stabilizing interactions of the A-TM1, A-TM2 and A-TM3 linkers, compared to the increased flexibility of these interactions in other P-type ATPases (17, 50, 51). In order for the A–P domain interaction to occur, the following changes take place: The A-domain moves toward the P-domain, the N-domain pivots about the N–P interface, away from the A-domain, and the P-domain makes a small movement (12, 19).

E2P state usually captures closely interacting ordered A- and P-domains, both of which are separated from the N-domain, thus making it relatively flexible (22). However, in the E2P state of Neo1, the A- and N-domains interact strongly, for a greater proportion of the reaction cycle, compared to other P4-ATPases (18).

### E2P-transition/E2-Pi-PL states

The E2P-transition state appears to be unmoving and mostly superimposable on the E2P structure (12). This intermediate, also captured by AlF_4_^-^, finds the A and P-domains bridged by AlF_4_^−^, and is associated with an A-domain rotation about the phosphorylation site, as compared to the E2P intermediate. This exposes the aspartylphosphate of the P-domain for a dephosphorylation reaction, with the glutamate of the DGET motif acting as a catalytic base. Since the A-domain is also linked to TM1 and 2, this shifts TM1-2 to open the substrate translocation pathway between TM1-4 and 6 (8) (Fig. 3*A*). This is triggered by the binding of a substrate lipid in the substrate entry site between TM1-4 and 6, which causes an allosteric change, resulting in the rotation of the A-domain around the phosphorylation site. This represents the substrate-dependent dephosphorylation of P4-ATPases.

AlF_4_^-^, in the presence of a substrate lipid, positions the cytosolic domains in the substrate-bound orientation (8) (Fig. 3*A*). The substrate can now enter the translocation pathway of the TM domains. Due to energy barrier associated with binding the hydrophilic head of the phospholipids, the transition from E2-Pi to E2-Pi-PL needs to overcome a huge energy potential of ∼34 kcal/mol (48). The shift of the A-domain between the E1P-ADP state and the E2P-PL state is only around 6 Å, unlike the 120˚ rotation observed in the case of ion transporters (17). The exoplasmic TM1-2 residues coordinate the PL headgroup. Further movement of PL along the pathway is accompanied by closure of the exoplasmic gate, through TM1-2 movement, moving the connected A-domain away from the P-domain. This creates an occluded state, where the substrate is completely buried within the membrane, inaccessible from both the cytosolic and exoplasmic leaflet (19). This causes substrate-dependent dephosphorylation and eventual substrate movement to the cytosolic leaflet (12). The substrate transport process is further elaborated in the next section. The transition from the E2 to E1 state is also accompanied by the increased flexibility of the N- and A-domains (12).

## Substrate recognition, translocation, and release through the TM domains

### Models for substrate translocation

**Figure 4:**
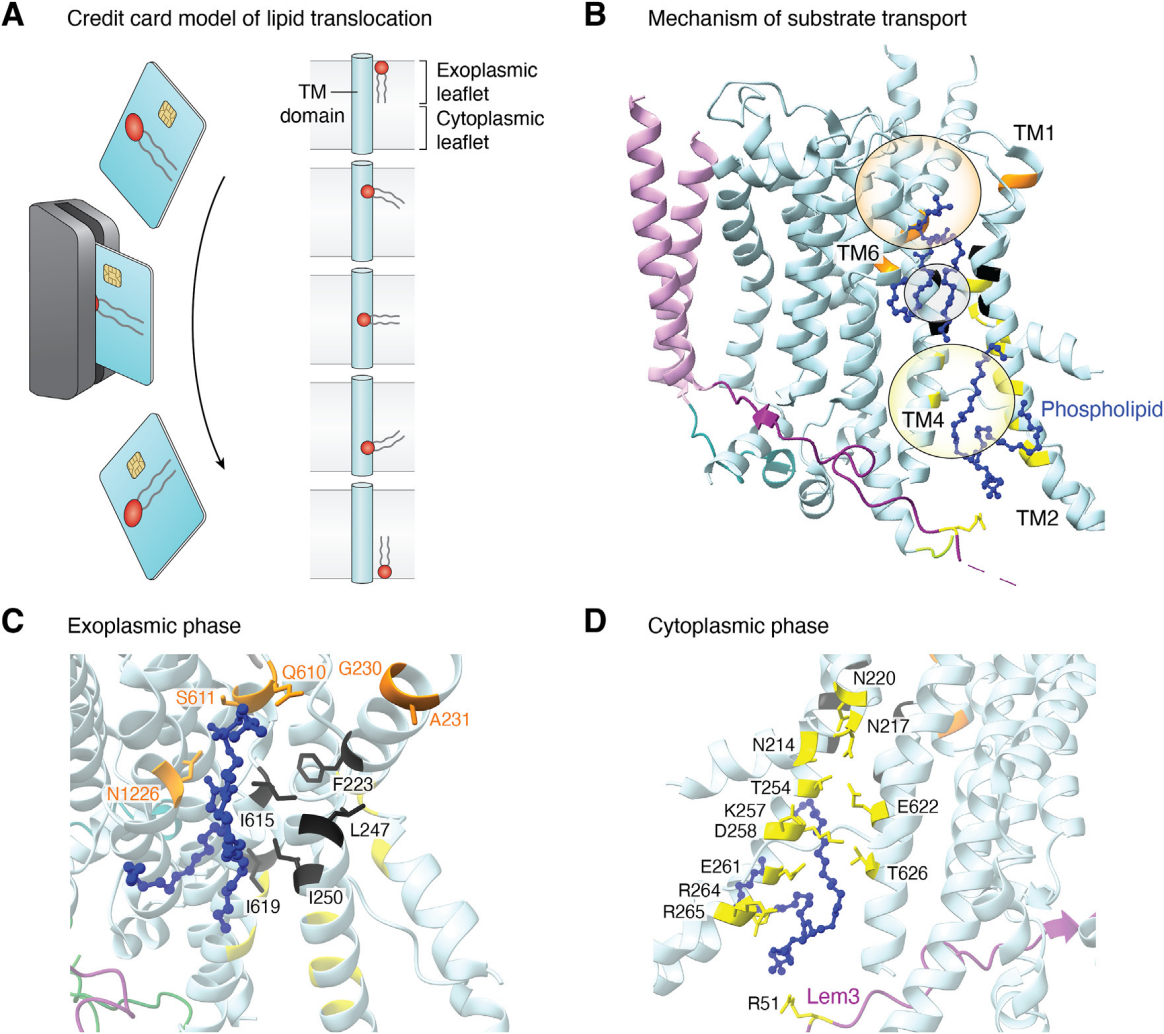
**Phospholipid translocation pathway.***A*, the credit card model. The phospholipid translocation pathway of P4-ATPases (*cyan*–α subunit; *purple*–β subunit) has been explained as being similar to the movement of a credit card through a reader, with the hydrophobic tails remaining in the membrane and the hydrophilic headgroup passing through the transmembrane domain in an ATP-dependent manner. This has been explained by different models including the two-gate model, the hydrophobic gate model, and the central cavity model. *B*, *C*, and *D*, Mechanism of substrate transport. The two-gate model identifies entry and exit sites (*orange residues*) for substrate selection. Meanwhile, hydrophobic gate model identifies a mechanism for substrate movement through two water-filled cavities (*orange and yellow circles*) separated by a hydrophobic gate (*black residues, black circle*), which prevents free movement of hydrophilic moieties such as water and the phosphate headgroup between them. Meanwhile, the central cavity model narrowed down on a large cavity between TM3, 4, and 5 as the phospholipid-binding site, where a TM4 tyrosine and phenylalanine determine specificity through cavity dimensions. Notably, cavity appears to be similar to the entry gate model and the presence of the large cavity could have been due to the use of a broad specificity flippase (42), thus making it similar to the hydrophobic gate model. The blue phospholipid is coordinated through the substrate translocation pathway between TM2, 4, and 6. Transport occurs in two phases, exoplasmic (*C*) and cytosolic (*D*), coupled to the Post-Albers reaction scheme. The phospholipid headgroup phospholipid headgroup initially binds at the exoplasmic entry gate, near G230 and A231 but is further selected at a deep site, involving S611, N1226, and surrounding residues (C) before the central hydrophobic gate, particularly I615 of the PISL motif, (*C*) before it is allowed into the cytosolic side to be released from the exit gate through a transient cytosolic exit site (*D*). TM, transmembrane.

Due to the presence of their hydrophobic tails and hydrophilic headgroups, phospholipids were proposed to flip through the membrane in a process akin to sliding a credit card through a card reader. This model, christened the ‘Credit card model’ (Fig. 4*A*), and subsequent molecular dynamics simulations have suggested that the hydrophobic lipid tails remain in the center of the bilayer throughout the transport cycle, with the headgroups moving 90˚ in each half of the transport cycle, through the TM domain of the P4-ATPases (52). The substrate specificities of P4-ATPases vary between the different members as listed in Table 1. Currently, three models of substrate translocation have been proposed to explain the molecular foundation of the credit card model, based on studies in different P4-ATPases (Fig. 4*B*).

**Table 1:** The substrate specificities of P4-ATPases from *Saccharomyces cerevisiae, Homo sapiens,* and *Arabidopsis thaliana*

Flippase	Substrates	References
ScDnf1	PC, GlcCer, GalCer, PS, lyso-PS, lyso-PE, lyso-PC	(74, 75, 78, 79)
ScDnf2	PC, PE, GlcCer, GalCer, PS, lyso-PS, lyso-PC, lyso-PE	(74, 75, 78, 79)
ScDnf3	PS, PC, PE	(80, 81)
ScDrs2	PS, PE	(63, 81, 82, 83)
ScNeo1	PE, PS, Lyso-PS	(18, 84)
HsATP8A1	PE, PS	(82, 85)
HsATP8A2	PE, PS	(58, 69, 82)
HsATP8B1	PE, PG, PI, PA, PS, PC, cardiolipin	(20, 21, 42, 60, 61, 78)
HsATP8B2	Plasmalogens	(86)
HsATP8B3	PS	(87)
HsATP8B4	Unknown	-
HsATP9A	Unknown	-
HsATP9B	Unknown	-
HsATP10A	PC, GlcCer	(61, 75)
HsATP10B	PC, GlcCer, GlcSph, SM	(75, 88)
HsATP10D	GlcCer	(75)
HsATP11A	PS, PE	(89, 90)
HsATP11B	PS	(91)
HsATP11C	PS, PE	(31, 92, 93, 94)
AtALA1	PS, PE	(95, 96)
AtALA2	PS	(97)
AtALA3	PC, PE, PS	(97, 98)
AtALA4	Unknown	-
AtALA5	PC, PE, SM	(99)
AtALA6	Unknown	-
AtALA7	Unknown	-
AtALA8	Unknown	-
AtALA9	Unknown	-
AtALA10	PC, PE, PS, PG, lyso-PC, SM, GlcCer	(55, 100)
AtALA11	Unknown	-
AtALA12	Unknown	-

GalCer, galactosylceramide; GlcSph, glucosylsphingosine; PG, phosphatidylglycerol; SM, sphingomyelin.

First, the two-gate model based on mutagenesis studies of Dnf1 postulates the existence of an exoplasmic entry gate and a cytoplasmic exit gate which together mediate selectivity for phospholipids (Fig. 4*B*). The entry gate includes exoplasmic part of TM1-2 and the luminal loop between TM1-2 and TM3-4, while the exit gate includes the cytosolic side of TM3 and 4 (53). Despite providing information on selectivity, this model does not detail the basis of movement between the two gates.

The hydrophobic gate model, based on mutagenesis and molecular dynamics studies in the bovine ATP8A2, identified the presence of two water-filled cavities between TM1, 2, 4, and 6, with a central hydrophobic belt of residues which prevent exchange of water and hence phospholipid movement between the two cavities (Fig. 4*B*). The hydrophobic Ile of the conserved PISL motif is critical for gating the hydrophilic phospholipid headgroup in the middle of the TM domain, while a nearby asparagine in TM4 was suggested to be hydrated and involved in PL transport (54). This motif, located in the TM4, houses a proline that is conserved across all P-type ATPases, generating a kink in the TM domains required for substrate transport. Due to its invariant nature in P-type ATPases, this proline is generally considered a point of reference for TM4 residues. This model specifically focuses on the transport mechanism, without venturing toward the arena of substrate selectivity.

The central cavity model is based on a homology model and chimeric mutagenesis of ALA10, a broad specificity plant flippase from *A. thaliana* (Fig. 4*B*). They identified a large central cavity in the TM domain, between TM3, 4, and 5 capable of holding a phospholipid molecule. This model suggests that the Ile of the conserved ^376^PISL^380^ motif is involved in regulating the cavity dimensions rather than directly coordinating the headgroup, while the nearby phenylalanine-374 (F374) is suggested to be the primary gating residue and regulates the microenvironment of the lipid headgroup along with tyrosine-373 (Y373). Unlike the other models, TM1-2 is suggested to be indirectly involved in the central cavity model (55). This model does not provide any theory on the selection of substrates by specific residues or their side chains, rather suggesting that most conserved residues affect the dimensions of the cavity for substrate translocation, despite using a broad specificity flippase.

Current structural literature has provided support for certain aspects of each of these models, as discussed below. Particularly, the pivotal role of TM4 in substrate transport remains a consensus in the available literature on P4-ATPases. Overall, it appears that the actual transport mechanism and pathway (Fig. 3) are a combination of all three models of substrate translocation.

### Structural insights into substrate transport

The roles of residues in the TM domains of P4-ATPases have been investigated through mutagenesis in great depth over the years, which has shed light on the substrate transport mechanism (Some examples of alanine scanning experiments include (54, 56, 57)). Recognition of the different phospholipids is suggested to be a combination of factors, including, but not limited to, the orientation and fit of headgroup and the acyl chain, substrate binding, and ability to induce dephosphorylation (22, 42, 52).

Exogenous or potentially endogenous substrate lipids have been identified at sites proximal to a GA/QQ motif of the entry gate (8, 12, 21) (Fig. 4, *B* and *C*). The PS headgroup is coordinated through hydrogen bonding with the PISL motif and surrounding hydrophilic residues of TM2, 4, and 6 (or nonpolar residues for PC flippases) (8). The PS-bound Drs2 structure finds the substrate bound at a site deep in the cavity between TM2, 4, and 6, with their molecular dynamics simulations hinting that substrate specificity is regulated there, rather than the entry gate (52). Meanwhile, the acyl chains have been identified near the substrate-binding pocket between TM2, 4, and 6, either proximal to TM2/4 or TM6/9/10 (8, 14, 19). Phospholipids protruding into the cytosolic leaflet have also been resolved proximal to the exit gate (12, 23) (Fig. 4*D*).

Notably, structures of ATP8B1 in the presence of substrates with different affinity revealed a similar binding and occlusion pattern but differences in interactions with the water network. These interactions are speculated to be involved in allosteric regulation of the cytosolic domains through TM2. Additionally, molecular dynamics simulations have also highlighted the importance of phospholipid-binding groove hydration in the process of substrate transport across the lipid bilayer (52). While the structures indicate that the PL headgroup replaces the water molecule in interacting with the PISL motif, the simulations suggested that the water molecules move with the PL as they approach this motif. This suggests that the role of water in substrate transport needs to be further investigated through other dynamic investigations such as simulations or nuclear magnetic resonance spectroscopy.

TM5-6 are involved in substrate transport in the ion transporting P-type ATPases, but these residues mostly hold accessory roles in the phospholipid transport of P4-ATPases (53). However, TM5 residues, such as K845 and N877 of bATP8A2, along with neighboring TM6 residues form a complex hydrogen bonding network, potentially associated with allosteric effects, positioning residues in TM4 and even the P/A-domains (53, 57, 58).

The role of the β-subunit in substrate translocation is still under discussion. The crystal structure of ATP11C found a PS molecule bound at a suggested exoplasmic loading site, consisting of the TM3-4 loop and CDC50 exoplasmic domain, leading into the TM PL transport cavity (19, 22, 23). There is still some debate about the legitimacy of this site, given that PS was loaded in excess of physiological concentrations (53).

The role of Lem3 (the β-subunit of both Dnf1 and Dnf2) in substrate binding was also captured in Dnf1, wherein mutations of Lem3-R51, part of the cytosolic gate, did not affect expression but altered GlcCer transport but not that of PC/PE (12) (Fig. 4*D*). The substrate exit gate of Drs2 also involved the cytosolic R151 of its β-subunit CDC50, analogous to R51 of Lem3 (15), while the monomeric Neo1 also possesses a cytoplasmic arginine which could compensate for the role of R51 (18).

Neo1, despite the absence of the β-subunit, appears to have a similar phospholipid-dependent ATP hydrolysis as characterized P4A-ATPases, with equivalents of substrate transport motifs of the latter but with variations which could be due to substrate specificity, monomeric nature, or some other feature of this group (18).

## **β-**subunit and its interaction with the catalytic subunit

The β-subunit of P4-ATPases has been established to be accessory but required for P4A/C-ATPases (59). (60, 61, 62). However, the association is not fixed and varies throughout the reaction cycle, with a regulatory role in the ATPase cycle (49, 60, 61, 62, 63, 64).

The β-subunit is primarily considered to be involved in stabilization and ER exit of the α-subunit to traffic it to their appropriate subcellular locations, although additional quality control mechanisms might also exist (65). They have been implicated in phosphoenzyme formation by ATP8B1 and ATP8B2 (66). As previously discussed, they are also implicated in substrate translocation (Fig. 4*D*).

The β-subunits are generally fewer in number than the α-subunits in each organism. *S. cerevisiae* has three β-subunits for five α-subunits, *H. sapiens* possess three for 14, while *A. thaliana* has five for 12. Additionally, the promiscuity of the interaction also varies—with *S. cerevisiae* exhibiting a relatively one-to-one interaction and *A. thaliana* an all-to-all interaction. Meanwhile, *H.sapiens* exhibit an interesting pattern, where most of the catalytic subunits interact with CDC50A, a few interact with CDC50B, and none are known to interact with CDC50C so far (60, 66).

Currently, the structures of HsCDC50A, HsCDC50B, ScLem3, ScCdc50, and *Chaetomium thermophilum* Cdc50 have been resolved through cryo-EM (12, 15, 16, 17, 20, 21). Despite their low sequence conservation of most β-subunits, they have similar structures and interactions with their corresponding α-subunits (15, 21). Although the cytosolic regions are usually poorly resolved due to flexibility, Lem3 and Cdc50 exhibited ordered N-terminal regions which interact electrostatically with the A-domain, potentially regulating it over the course of the reaction cycle (12, 17). The N-terminal regions have been implicated in transport. α-subunit interactions occur in the cytosolic, exoplasmic, and TM regions through the helices and the regions linking them (8, 15, 20, 21, 67, 68).

β-subunits generally possess two conserved features which are essential for stabilizing the α-subunit—glycosylation and intrachain disulfide bond formation. Altering the glycosylation affects the expression and function, while some of the α-subunit mutations impacted glycosylation but not activity (22, 67, 69, 70, 71). The β-subunits were generally found to have two intrachain disulfide bonds, which are necessary for complex formation, with the C-terminal bond being more important (8, 12, 15, 68). Although certain additional cysteines were present in the TM domain, they were not oriented toward each other (16) but could be forming a bond with the α-subunit (13).

## Interaction with lipid bilayers

Nanodiscs are used to study the behavior of membrane proteins in the lipid bilayer by embedding them in a large number of phospholipids held together by an amphipathic membrane scaffold protein (72). The structures of ScDnf1, CtDnf1, and hATP11C reconstituted with nanodiscs have been resolved by cryo-EM (13, 14, 23). The Dnf1 structures have been resolved for the E1-ATP and E2P states, while the ATP11C structures are in E1P and E2-Pi-PL states.

The structures of CtDnf1 (from *C. thermophilum*) and ScDnf1 have both been reconstituted in yeast polar lipids but with membrane scaffold proteins of different lengths (13, 14). Some of these structures exhibited a downward and separated A-domain, unlike that seen in detergents. The resulting A-domain interaction with the lipids of the nanodisc was attributed to a positively charged patch, which was also observed in detergent structures, although not proximal to the membrane in the latter. Considering the absence of this patch in PS flippases, this was suggested to be a PC flippase–specific feature to interact with membrane phospholipids (13, 14).

A change in the arrangement of the TM helices was also observed in reconstituted ScDnf1. This structure had a straight TM4 helix, in contrast to the characteristic kinked TM4 due to a conserved proline of P-type ATPases, an essential requirement for substrate translocation. Further, TM1 and 2 had exchanged their positions (54). This uncharacteristic TM domain arrangement was proposed to be a resting state to protect against constitutive activation of the PC flippase Dnf1 in PC-rich membranes. Consequently, when the same states were reconstituted in nanodiscs consisting of 90% PS and 10% PC, some particles had upright A-domains and deformed TM4, as seen in other detergent structures. Thus, the membrane compositions were suggested to be involved in regulating the activity of Dnf1 (13).

For ATP11C reconstitution, the phosphate analogs AlF and BeF behaved differently compared to the detergent samples. A large proportion of the AlF-treated particles had PS occluded, although other structural features resembled the E1-ATP state. On the other hand, the BeF-treated sample was found to drive the transport cycle further than the conventional E2P state to generate an exoplasmic gate-closed E2-Pi conformation (23).

The nanodisc structures also offer insight into the thickness of the membrane around the protein. The CtfDnf1 E1-ATP structures and BeF-ATP11C structures identified decreased exoplasmic and increased cytosolic thickness, respectively (14, 23). These are suggested to be phospholipid loading sites at the time of entry and exit. A similar membrane observation was also made using molecular dynamics simulations (52).

Some additional studies have been conducted in the past to determine the influence of the membrane in regulating the activity of P4-ATPases. One such study found that increased membrane tension due to hypotonic conditions is associated with reduced lipid translocation (73). Additionally, exogenous addition of substrate and nonsubstrate phospholipids differentially alters the ability of Dnf2 to translocate the fluorescently labeled nitrobenzoxadiazole PC and nitrobenzoxadiazole-GlcCer in cellular transport assays. This appears to be due to the impact on membrane curvature as well as crowding and competition at the entry gate. However, this remains to be studied further in other flippases. Moreover, the sterol content of the membrane has also been shown to have a modulatory effect on transport (74).

## Outlook and future perspectives

Currently, several members of the P4-ATPase family have been characterized using different methods, providing insight into their structure-function relationships. Earlier, the giant-substrate problem of P4-ATPases, *i.e.*, the ability of P4-ATPases to transport large phospholipids, unlike their ion-transporting relatives, was one of the biggest enigmas in the field. Currently, through experimental and computational approaches, the mechanisms involved in transporting these giant phospholipids have become clearer.

However, several other questions regarding these transporters still remain to be solved. For instance, the exoplasmic part of the transport pathway has been biochemically characterized by several studies, but the cytoplasmic part, although possibly characterized by structural approaches, remains to be functionally studied in-depth. Additionally, although the path of lipid transport has been studied, the basis of specificity, especially between sphingolipids, is still largely unknown. Although the GlcCer preference of certain P4-ATPases has been studied (75, 76), the basis of selection for or against another sphingolipid, sphingomyelin, by P4-ATPases is not fully known. This particularly requires additional studies involving broad specificity flippases from *A. thaliana.*

Further, the roles of TM7-10 are not very well understood. Generation of TM7-10 chimeric mutants between Drs2 and Dnf1 produced misfolded proteins which were localized to the ER (77). TM7-10, particularly TM10 is proximal to the β-subunit and thus misfolding of chimeric mutants might be due to interference with this interaction. Alternatively, this could also be affecting the autoregulatory interaction with the C-terminal or regulatory lipids (16). Meanwhile, TM9 has been implicated in acyl chain binding and an alternate phospholipid translocation pathway in structural literature (19). Notably, an alternate access mechanism involving TM9 has also been hinted at, but the requirement for and behavior of this pathway is unclear. Thus, further investigation into the roles of these regions is required.

Although some membrane-mimetic structures have been solved, there remains to be a uniform investigation characterizing the behavior of P4-ATPases in membrane-mimetic systems. This is required to understand the regulation of their structure and function by membrane properties such as curvature and composition, through the use of artificial lipid bilayers of known composition such as nanodiscs and proteoliposomes. This is crucial to understand their functional mechanisms while being embedded in a membrane of their substrates. Additionally, it will also shed light on the regulatory role of nonsubstrate lipids. Further, the autoregulatory mechanisms of several members of this family remain to be characterized.

Several mysteries also exist regarding the P4B group of proteins, such as their ability to exist as monomers, their substrate specificity, and similarity to the P4C group. Moreover, although there is some insight into the posttranslational modifications of the P4A group, there is no insight into that of the P4B group yet. Further, a systematic investigation of the posttranslational modification profiles of P4-ATPases is yet to be performed.

In addition to this, several P4-ATPases have been suggested to interact with other proteins, especially in the context of vesicle formation. These complexes have yet to be structurally resolved. This would provide further context on the transient nature of the interactions with the β-subunit and the stabilization of the catalytic α-subunit by different interactors. As such, several further biochemical, biophysical, structural, and computational studies are required to further characterize the functional mechanisms of this family of proteins.

## Conflict of interest

The authors declare that they have no conflicts of interest with the contents of this article.
